# Post-intervention durability of standalone digital behavior change interventions for physical activity in adults: a focused mini review and quantitative synthesis

**DOI:** 10.3389/fpubh.2026.1878053

**Published:** 2026-07-27

**Authors:** Jian Zeng, Hansen Li, Xing Zhang

**Affiliations:** 1 Mianyang Teachers’ College, Mianyang, Sichuan, China; 2 School of Physical Education, Sichuan Agricultural University, Ya'an, China; 3 Department of Physical Education and Sport, Faculty of Sport Sciences, University of Granada, Granada, Spain

**Keywords:** digital interventions, exercise, mobile applications, physical activity, wearable devices

## Abstract

**Background:**

Standalone digital behavior change interventions (DBCIs) are increasingly used to promote physical activity (PA), but their post-intervention durability remains unclear. This focused mini review with quantitative synthesis examined maintenance and attenuation of standalone DBCI effects on PA in adults.

**Methods:**

PubMed, Web of Science Core Collection, and SPORTDiscus were searched without date restrictions, with the final search conducted on 3 May 2026. Eligible studies were randomized controlled studies involving adults, standalone DBCIs targeting PA, a comparator not receiving the target structured DBCI, and PA outcomes assessed post-intervention and at ≥1 follow-up. Random-effects meta-analyses used Hedges’ g, with the longest follow-up used for the primary maintenance analysis.

**Results:**

Seventeen studies involving 8,990 participants were included in the review, of which 13 contributed effect sizes to the primary quantitative synthesis. Standalone DBCIs showed a small, non-significant effect at the longest follow-up (*g* = 0.13; *p* = 0.066; *I*^2^ = 64.2%), with low-certainty evidence. Time-window analyses showed a small significant post-intervention effect (*g* = 0.14; *p* = 0.048), whereas follow-up effects were small and non-significant. Meta-regression showed no clear progressive attenuation.

**Conclusion:**

Standalone DBCIs showed small positive post-intervention point estimates, but evidence remains insufficient to establish reliable maintenance after active support is reduced or withdrawn. Future studies should use repeated follow-ups and report engagement, continued tool access, and post-intervention support.

## 1 Introduction

Regular physical activity (PA) is a major modifiable determinant of health across adult populations (1, 2). A large body of evidence indicates that higher PA levels are associated with reduced risks of all-cause mortality, cardiovascular disease, type 2 diabetes, several cancers, and depression, as well as better physical functioning and overall well-being (2–6). Nevertheless, insufficient PA remains a major and growing global challenge (7, 8). A recent pooled analysis of population-based surveys across 197 countries and territories showed that the global prevalence of insufficient PA among adults increased from 23.4% in 2000 to 31.3% in 2022, corresponding to approximately 1.8 billion adults not meeting recommended activity levels (7). Given this persistent and increasing burden, identifying effective, scalable, and sustainable strategies to support PA participation in adults remains an important public health priority.

To address the growing burden of insufficient PA, considerable efforts have therefore been devoted to developing effective PA promotion strategies (9, 10). Among these, digital behavior change interventions (DBCIs) have been proposed as a promising and potentially scalable approach (11–13). DBCIs use digital technologies, such as websites, mobile applications, wearable devices, or social media platforms, to support or facilitate behavior change (11, 14). For PA promotion, they can deliver behavior change techniques such as goal setting, self-monitoring, feedback on performance, prompts or reminders, action planning, and social support through automated or minimally supported digital systems (11, 15). Compared with traditional face-to-face interventions, DBCIs may offer broad reach, flexible access, relatively low delivery costs, and tailored or personalized feedback based on users’ behavior or self-reported data (11, 12, 14). These features have generated increasing interest in DBCIs as a scalable strategy for addressing insufficient PA in adults (11, 12, 14).

In recent years, increasing evidence has examined the effects of DBCIs on PA across different adult populations and digital delivery formats (16–18). However, findings across individual studies have not been entirely consistent. For example, Van Dyck et al. (19) reported positive or borderline intervention effects on accelerometer-measured PA and improvements in several self-reported PA outcomes among older adults. In contrast, Collombon et al. (20) found no significant intervention effects on either self-reported or accelerometer-measured moderate-to-vigorous physical activity among adults aged 50 years and older. These mixed findings make it difficult to determine the overall impact of DBCIs on PA and highlight the need for evidence synthesis.

To clarify inconsistent findings from individual studies, several systematic reviews and meta-analyses have synthesized evidence on digital interventions for PA promotion (21, 22). However, many earlier syntheses included interventions that combined digital components with face-to-face counseling, supervised exercise, printed materials, or broader lifestyle programs, making it difficult to isolate the specific contribution of standalone DBCIs (21, 22). More recently, Lee and Park (23) focused specifically on standalone DBCIs and found a significant small-to-moderate effect on PA in adults. Nevertheless, their review primarily focused on post-intervention effects, leaving unclear whether PA improvements persist after active intervention support ends and whether effects attenuate over time. Therefore, this focused mini review with quantitative synthesis aimed to evaluate the post-intervention durability and attenuation of standalone DBCI effects on PA in adults.

## 2 Methods

### 2.1 Registration and search strategy

This systematic review and meta-analysis was conducted following the Cochrane Handbook and PRISMA 2020 guidance (24, 25), and the protocol was prospectively registered in PROSPERO in May 2026 (CRD420261384215). PubMed, Web of Science Core Collection, and SPORTDiscus were searched without date restrictions, with the final search conducted on 3 May 2026, using terms related to physical activity, sedentary behavior, and standalone digital behavior change interventions. Full search strategies are provided in Supplementary Table 1. Additional records were identified through Google Scholar, reference lists of included studies, and relevant reviews. All records were managed in EndNote 21, where duplicates were removed automatically and verified manually.

### 2.2 Eligibility criteria

Eligibility criteria were defined using the PICOS framework.

Population: Adults aged 18 years or older.

Intervention: Standalone DBCIs targeting physical activity.

Comparison: A control or comparison condition that did not receive the target structured DBCI.

Outcomes: At least one PA outcome assessed at the post-intervention assessment and at least one subsequent follow-up assessment.

Study design: Randomized controlled studies.

We included peer-reviewed journal articles published in English. In this review, standalone DBCIs were defined as interventions primarily delivered through digital technologies, such as websites, mobile apps, text messaging, activity trackers, or other online platforms, and including at least one structured behavior change technique delivered digitally (23). Eligible comparators were conditions that did not receive the target structured DBCI, including wait-list/no-intervention controls, usual care or minimal PA education, and static digital information without structured behavior change techniques. Studies had to report at least one PA outcome at the post-intervention assessment and at least one subsequent follow-up assessment. Eligible PA outcomes included moderate-to-vigorous physical activity, total physical activity, walking, step counts, and PA frequency or duration, assessed using device-based or questionnaire-based methods. When multiple eligible outcomes were reported, one outcome was selected according to a prespecified hierarchy: device-measured moderate-to-vigorous physical activity, self-reported moderate-to-vigorous physical activity, total physical activity, step counts, and walking or domain-specific physical activity. When multiple follow-up assessments were available, the longest follow-up was used for the primary maintenance analysis, while other time points were retained for time-window and attenuation analyses. Studies meeting the review eligibility criteria were included in the qualitative synthesis. For the primary quantitative synthesis, studies also had to provide extractable and methodologically compatible data for calculating the prespecified between-group effect size.

### 2.3 Study selection and data extraction

All records were imported into EndNote 21 for reference management, and duplicates were removed using automatic matching followed by manual verification of DOI, journal, and pagination. Two reviewers (XZ and HSL) independently screened titles, abstracts, and full texts against the prespecified eligibility criteria. Disagreements were resolved through discussion or, when necessary, adjudication by a third reviewer (JZ).

Data were extracted using a standardized Excel sheet. Extracted information included study identification, design, sample size, participant characteristics, intervention and comparator characteristics, intervention duration, post-intervention and follow-up time points, PA outcomes, measurement methods, and quantitative data required for effect-size calculation. Two reviewers (JZ and XZ) independently extracted the data, with disagreements resolved through discussion or consultation with a third reviewer (HSL). When numerical data were available only in figures, values were digitized using GetData Graph Digitizer v2.26.

### 2.4 Assessment of bias and evidence quality

Risk of bias was assessed using the revised Cochrane risk of bias tool for randomized trials (RoB 2) (26). The assessment covered five domains: bias arising from the randomization process, bias due to deviations from intended interventions, bias due to missing outcome data, bias in measurement of the outcome, and bias in selection of the reported result. Each domain was judged as “low risk of bias,” “some concerns,” or “high risk of bias,” and an overall risk of bias judgment was assigned for each study. Two reviewers (XZ and HSL) independently conducted the assessments. Disagreements were resolved through discussion, with a third reviewer (JZ) consulted when necessary.

The certainty of evidence for each pooled outcome was assessed using the Grading of Recommendations Assessment, Development and Evaluation (GRADE) approach (27). Evidence from randomized controlled studies was initially rated as high certainty and could be downgraded by one or two levels based on risk of bias, imprecision, inconsistency, indirectness, and publication bias when applicable. The final certainty of evidence was classified as high, moderate, low, or very low.

### 2.5 Statistical analysis

Meta-analyses were conducted in R version 4.3.1 using the metafor and clubSandwich packages (28–30). For the purpose of this review, intervention end was operationalized as the scheduled end of active structured DBCI delivery in each original trial. Assessments after this point were considered post-intervention or follow-up assessments for the durability analyses. Because some studies may have allowed continued passive access to websites, apps, wearable feedback, automated reminders, or previously delivered digital materials after active delivery ended, post-intervention digital access and engagement/adherence reporting were extracted separately and summarized in Supplementary Table 7. The standardized mean difference was expressed as Hedges’ *g*, with positive values favoring the standalone DBCI. For the primary maintenance analysis, one effect size per study was retained according to the prespecified outcome hierarchy and the longest available follow-up after the post-intervention assessment. Studies that met the review eligibility criteria but did not provide extractable effect sizes compatible with this primary approach were retained in the qualitative synthesis and, where possible, examined in sensitivity analyses. Random-effects models fitted with restricted maximum likelihood estimation were used to estimate pooled effects (25). Maintenance was defined as the persistence of between-group effects at follow-up after the active intervention period had ended. The Hartung–Knapp adjustment was applied, and effects were reported with 95% confidence intervals and, where applicable, 95% prediction intervals (25, 31). Heterogeneity was quantified using *I*^2^ and *τ*^2^. To examine changes over time, effect sizes were classified as post-intervention, short-term follow-up (>0 to 3 months after intervention end), medium-term follow-up (>3 to 6 months), or long-term follow-up (>6 months). Because some studies contributed multiple time points, multilevel random-effects models with cluster-robust variance estimation were used (30). Attenuation was examined using meta-regression, with months after intervention end entered as a continuous moderator. Sensitivity analyses tested alternative analytic assumptions, and small-study effects were assessed using funnel plots and Egger’s regression test when appropriate (32). All tests were two-sided, with *p* < 0.05 considered statistically significant.

## 3 Results

### 3.1 Study selection

The study selection process is provided in Supplementary Figure 1. The database search identified 11,987 records, of which 7,018 remained after duplicate removal. After title and abstract screening, 38 reports were sought for retrieval; two could not be obtained despite attempts to contact the corresponding authors (33, 34). The remaining 36 full-text reports were assessed for eligibility, with 19 excluded because of ineligible comparator conditions, ineligible or non-isolatable interventions, or no eligible follow-up assessment after the post-intervention assessment. Supplementary searches identified no additional eligible reports.

### 3.2 Study characteristics

Seventeen randomized controlled studies were included, comprising 8,990 participants, with sample sizes ranging from 46 to 2,330 (16–20, 35–46). The included studies involved general/non-clinical adult populations as well as clinical or health-risk adult populations, including cancer survivors (18, 35, 41), adults with knee and/or hip osteoarthritis (45), and adults with overweight or obesity (36–38). Intervention duration ranged from a brief single-session online task to 6 months, and follow-up assessments after the post-intervention assessment ranged from several weeks to 12 months (16–20, 35–46). Most interventions were web-based, with the remaining studies using mobile-, wearable-, or mixed digital delivery formats. Comparator conditions did not receive the target structured DBCI. PA outcomes included moderate-to-vigorous PA, total PA, walking, step counts, PA frequency or duration, and domain-specific PA, assessed using self-report questionnaires, device-based measures, or both. The characteristics of the included studies and their contribution to the primary quantitative synthesis, qualitative synthesis, or sensitivity analyses are detailed in Supplementary Table 2; briefly, Unick et al. (35) and Duan et al. (42) lacked compatible numerical data, Joseph et al. (36) used an active digital comparator, Myers et al. (38) reported an adjusted path-model effect, and Peels et al. (46) contributed its web-based basic arm to the primary model. Post-intervention access and engagement reporting varied across studies and are summarized in Supplementary Table 7. In several trials, follow-up assessments clearly occurred after active structured delivery ended, but complete withdrawal of all digital tools or previously delivered digital materials was often not reported or remained unclear. Engagement metrics were reported inconsistently and included website or app use, lesson or module completion, device wear, step logging, challenge completion, retention, or satisfaction, depending on the intervention.

### 3.3 Risk of bias assessment

The risk-of-bias assessment for all 17 studies included in the review is provided in Supplementary Figure 2. Overall, one study was judged as low risk of bias, 10 as having some concerns, and six as high risk of bias. Common concerns involved incomplete reporting of randomization or prespecified analyses, missing outcome data, and outcome measurement. High attrition contributed to risk of bias in the missing data domain, while self-reported physical activity outcomes increased concerns about measurement bias. Because participant blinding is generally infeasible in digital behavior change trials, lack of blinding alone was not judged as high risk, but it was considered when outcomes were self-reported.

### 3.4 Meta-analysis

**Figure 1:**
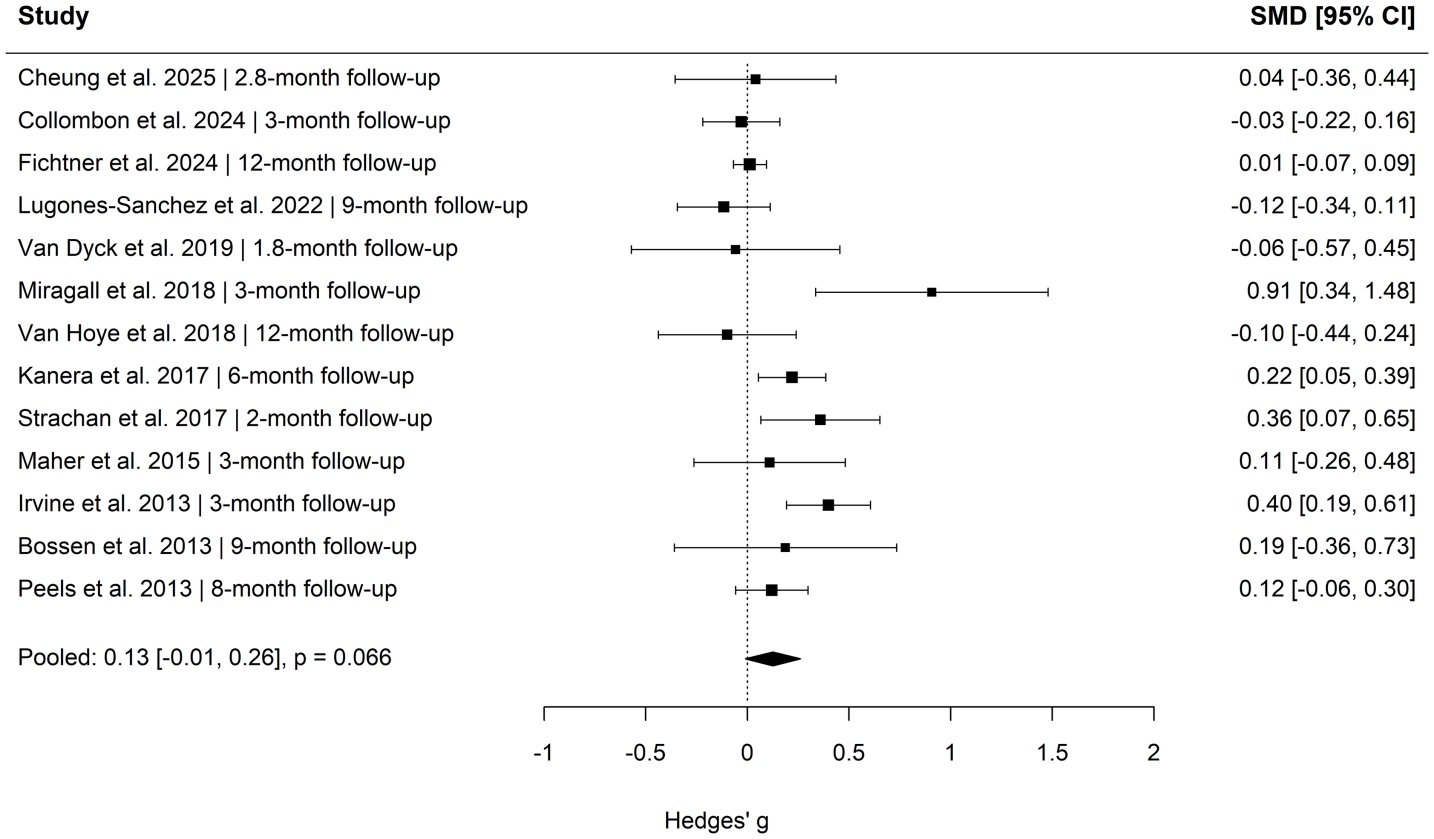
Forest plot of the effect of standalone digital behavior change interventions on physical activity at the longest follow-up after the post-intervention assessment among the 13 studies contributing to the primary quantitative synthesis.

Among the 13 studies contributing to the primary quantitative synthesis, standalone DBCIs showed a small positive but non-significant effect on PA at the longest follow-up after the post-intervention assessment (Hedges’ *g* = 0.13; *p* = 0.066; Figure 1 and Table 1). Heterogeneity was moderate to substantial (*I*^2^ = 64.2%), and the prediction interval suggested that effects may vary across future studies. The certainty of evidence for this primary maintenance outcome was low, mainly because of risk of bias and imprecision.

**Table 1:** Summary of meta-analytic findings for physical activity outcomes.

Outcome	Effects/studies	Hedges’ *g*	95% CI	*p*-value	*I* ^2^
Longest follow-up after the post-intervention assessment	13/13	0.13	−0.010 to 0.260	0.066	64.2%
Post-intervention	9/9	0.14	0.001 to 0.281	0.048	–
Short-term follow-up (>0–3 months)	10/8	0.16	−0.012 to 0.333	0.064	–
Medium-term follow-up (>3–6 months)	3/3	0.15	−0.039 to 0.336	0.098	–
Long-term follow-up (>6 months)	5/5	0.13	−0.002 to 0.264	0.052	–

Effects/studies indicates the number of effect sizes and the number of unique studies contributing to each analysis. Some studies contributed more than one eligible time point within the same time window; these statistically dependent effects were handled using multilevel models with cluster-robust variance estimation. For the time-window analyses, row-specific *I*^2^ values were not estimated because estimates were obtained from a single multilevel time-course model. The approximate model-level I^2^ for the time-course model was 51.3%.

Across time windows, pooled effects remained small. A statistically significant effect was observed at the post-intervention assessment (*g* = 0.14; *p* = 0.048), whereas effects did not reach statistical significance at short-term (*g* = 0.16; *p* = 0.064), medium-term (*g* = 0.15; *p* = 0.098), or long-term follow-up (*g* = 0.13; *p* = 0.052). GRADE assessment indicated moderate-certainty evidence for the post-intervention effect and low-certainty evidence for all follow-up effects.

Meta-regression showed no clear evidence of progressive attenuation over time, either when all available time points were included (*β* = −0.001 per month; *p* = 0.790) or when only follow-up assessments were analyzed (*β* = −0.01 per month; *p* = 0.196). Intervention duration was also explored as a moderator, but no clear association with maintenance effects was observed in either the unadjusted model (*β* = −0.003 per week; *p* = 0.740) or the model adjusted for follow-up duration (*β* = −0.008 per week; *p* = 0.510). Exploratory subgroup analyses showed no statistically significant differences by population context or PA measurement method. Sensitivity analyses yielded similar small and directionally consistent effects, and Egger’s regression test was not statistically significant, although this finding should be interpreted cautiously because the test was based on only 13 studies. Detailed meta-regression, subgroup, sensitivity, and small-study-effect analyses are provided in Supplementary Tables 3–6 and Supplementary Figures 3–5.

## 4 Discussion

To our knowledge, this is the first systematic review and meta-analysis to examine the post-intervention maintenance and attenuation of standalone DBCIs for promoting physical activity (PA) in adults. Overall, point estimates tended to favor standalone DBCIs across the post-intervention and follow-up assessments, but the primary maintenance effect at the longest available follow-up did not reach statistical significance. Effects were small across all time windows, with only the post-intervention effect reaching statistical significance. Meta-regression showed no clear evidence that effects attenuated as follow-up duration increased. The certainty of evidence was moderate for the post-intervention effect but low for the primary maintenance and follow-up effects. From a practical perspective, the findings indicate small positive point estimates at the post-intervention assessment, but current evidence remains insufficient to establish reliable maintenance after active intervention support is reduced or withdrawn. For public health practice, standalone apps, web platforms, and wearable-supported interventions should therefore be interpreted as potentially promising but not yet proven tools for sustained PA behavior change. The practical meaning of these small standardized effects is also uncertain because the included studies used heterogeneous PA outcomes, preventing reliable translation into a single metric such as steps/day or minutes/week of MVPA.

### 4.1 Overall maintenance effects and attenuation over follow-up

The time-window analysis showed a small but statistically significant effect of standalone DBCIs on PA at the post-intervention assessment. This finding is broadly consistent with Lee and Park (23), who reported that standalone DBCIs improved PA in adults. However, the effect observed in the present review was smaller, likely because our review focused specifically on post-intervention maintenance and included only studies reporting both post-intervention and subsequent follow-up PA outcomes. This narrower eligibility criterion resulted in a smaller and different evidence base. The broader issue of long-term maintenance may also be relevant to emerging AI-supported digital health tools, such as conversational agents, virtual coaches, and chatbots; however, these tools were not analyzed as a separate intervention category in the present review, and the current evidence base remains limited by insufficient extended follow-up (47–50).

Importantly, post-intervention follow-up did not necessarily indicate complete withdrawal of all digital access. As summarized in Supplementary Table 7, post-intervention access to websites, apps, wearable feedback, reminders, or previously delivered materials was often unclear, and engagement/adherence metrics were inconsistently reported. Therefore, the pooled estimates should be interpreted as durability after active structured delivery ended rather than definitive evidence of maintenance after complete technology withdrawal; small effects may reflect limited intervention efficacy, declining engagement over time, or both. Evidence for maintenance after the active intervention period was weaker. The primary maintenance analysis showed a small positive effect at the longest follow-up, but this effect did not reach statistical significance. Similarly, effects remained small and non-significant across short-, medium-, and long-term follow-up windows. The certainty of evidence also supports cautious interpretation, with moderate-certainty evidence for the post-intervention effect but low-certainty evidence for the maintenance and follow-up effects. Thus, current evidence shows small positive point estimates at the post-intervention assessment, but remains insufficient to establish reliable maintenance after active intervention support is reduced or withdrawn. Several factors may explain this pattern. Standalone DBCIs often depend on continued engagement with self-monitoring, feedback, goal setting, prompts or reminders, and action planning (14, 15, 51). These components may support PA while the intervention is active, but their influence may weaken when reminders stop, feedback becomes less frequent, or users disengage from the digital platform (14, 15, 51, 52). In addition, some comparator conditions, such as written PA recommendations, newsletters, brief counseling, or static digital health information, may have promoted PA to some extent, thereby reducing between-group differences at follow-up (16, 18, 35, 37).

Importantly, the small and broadly similar point estimates across time windows should not be interpreted as clear evidence of maintenance. Rather, the current evidence is too imprecise to confirm either reliable maintenance or progressive attenuation. Consistently, meta-regression showed no clear evidence that effects declined as follow-up duration increased; however, this does not demonstrate stability over time. Small and imprecise effects, limited studies in some follow-up windows, and variable follow-up schedules may have reduced the ability to detect temporal patterns (25, 53). Future studies should incorporate repeated follow-up assessments and further examine whether maintenance-oriented strategies, such as habit formation, relapse prevention, adaptive goal revision, booster sessions, continued self-monitoring, and sustained automated feedback, can improve the durability of PA effects after active intervention support is reduced or withdrawn.

### 4.2 Heterogeneity and exploratory subgroup findings

The primary maintenance analysis showed moderate to substantial heterogeneity, suggesting that the effects of standalone DBCIs varied across studies. This variability may reflect differences in study design, intervention characteristics, measurement methods, follow-up timing, intervention duration, digital modality, continued digital access, and participant engagement. Exploratory subgroup analyses showed no clear evidence that maintenance effects differed by population context or PA measurement method. Specifically, effects did not differ significantly between general/non-clinical adult populations and clinical or health-risk adult populations, or between self-reported and device-measured PA outcomes. However, these findings should be interpreted cautiously because the number of studies within each subgroup was limited. We considered subgroup analyses by intervention modality. However, given the focused scope of this mini review and quantitative synthesis, the limited number of studies contributing to the primary model, and the overlapping or multicomponent nature of several digital modalities, a formal modality-based subgroup analysis was not considered sufficiently stable or clinically meaningful. Instead, intervention modality, intervention duration, post-intervention access, and engagement/adherence reporting are summarized at the study level in Supplementary Table 7. As more evidence becomes available, future meta-analyses should re-examine whether digital modality, continued access, intervention duration, and engagement patterns moderate post-intervention maintenance.

### 4.3 Strengths and limitations

This review has several strengths, including its specific focus on standalone DBCIs, its emphasis on post-intervention maintenance and attenuation rather than immediate effectiveness alone, and its use of time-window analyses and GRADE assessment to support cautious interpretation. However, several limitations should be acknowledged. First, because this review focused on adults, the findings may not be generalizable to other populations, such as children and adolescents. Second, although exploratory subgroup analyses were conducted, the limited number of eligible studies restricted these analyses to population context and PA measurement method; therefore, they could not fully explain the sources of heterogeneity. Third, the certainty of evidence was limited by the relatively small number of studies, especially within some follow-up windows, and by the risk of bias in many included studies. Consequently, the maintenance findings should be interpreted cautiously. Fourth, although Egger’s regression test was not statistically significant, it was based on only 13 studies and was likely underpowered; therefore, this result should not be interpreted as definitive evidence that publication bias or small-study effects were absent. Finally, many trials reported only one follow-up assessment after the post-intervention assessment and provided limited information on intervention engagement, continued use of digital tools, and post-intervention support, restricting our ability to fully characterize maintenance and attenuation trajectories after active intervention support was reduced or withdrawn.

## 5 Conclusion

The current systematic review and meta-analysis examined the post-intervention maintenance and attenuation of standalone DBCIs for promoting PA in adults. Standalone DBCIs showed a small positive effect on PA at the post-intervention assessment, whereas effects at subsequent follow-up assessments remained small and did not reach statistical significance. The primary maintenance effect at the longest follow-up was also small, non-significant, and supported by low-certainty evidence. Therefore, although no clear progressive attenuation was observed, the current findings should be interpreted primarily as small positive point estimates rather than conclusive evidence of durable PA improvement. Current evidence remains insufficient to establish reliable maintenance after active intervention support is reduced or withdrawn. Thus, standalone DBCIs should not yet be viewed as proven long-term maintenance solutions. Future studies would benefit from repeated follow-up assessments and clearer reporting of intervention engagement, continued digital tool access, and post-intervention support.
