# Supplementary material for: Post-intervention durability of standalone digital behavior change interventions for physical activity in adults: a focused mini review and quantitative synthesis
Source: Front Public Health. 2026 Jul 27;14:1878053. doi: 10.3389/fpubh.2026.1878053 (PMC13454421; doi:10.3389/fpubh.2026.1878053)
Supplement: Supplementary file 1 [file Supplementary_file_1.DOCX]

Supplementary Material

# 1 Supplementary Figures and Tables

## 1.1 Supplementary Figures

Supplementary Figure 1. Study selection flow diagram of included and excluded research.

Supplementary Figure 2. Risk-of-bias assessment for all 17 studies included in the review using the revised Cochrane risk-of-bias tool for randomized trials.

**Supplementary Figure 3. Bubble plot of attenuation trend over follow-up duration based on analyzable time-course effect sizes.**

**Supplementary Figure 4. Funnel plot for small-study effects based on the 13 studies contributing to the primary quantitative synthesis.**

**Supplementary Figure 5. Time-course effects of standalone DBCIs on physical activity across post-intervention and follow-up windows.**

## 1.2 Supplementary Tables

Table 1. The specific search strategies for each database.

| Database | Step | Search strategies | Number | Date |
| --- | --- | --- | --- | --- |
| Web of Science | #1 | TI=("physical activity" OR walking OR steps OR "step count* "OR sedentary* OR "sedentary behavio*" OR sitting) | 6156 | 2026/05/03 |
|  | #2 | TS=("digital behavio*" OR "digital intervention*" OR "web-based intervention*" OR "internet-based intervention*" OR "online intervention*" OR eHealth OR mHealth OR "mobile app intervention*" OR "smartphone app intervention*" OR "mobile phone intervention*" OR "text messag* intervention*" OR "SMS intervention*" OR "wearable electronic device*" OR "activity tracker*" OR "fitness tracker*" OR Fitbit* OR pedometer*) |  |  |
|  | #3 | #1 AND #2 |  |  |
| SPORTDiscus | #1 | TI ("physical activity" OR walking OR steps OR "step count*" OR sedentary* OR "sedentary behavio*" OR sitting) | 1346 | 2026/05/03 |
|  | #2 | ( TI ("digital behavio*" OR "digital intervention*" OR "web-based intervention*" OR "internet-based intervention*" OR "online intervention*" OR eHealth OR mHealth OR "mobile app intervention*" OR "smartphone app intervention*" OR "mobile phone intervention*" OR "text messag* intervention*" OR "SMS intervention*" OR "wearable electronic device*" OR "activity tracker*" OR "fitness tracker*" OR Fitbit* OR pedometer*) OR AB ("digital behavio*" OR "digital intervention*" OR "web-based intervention*" OR "internet-based intervention*" OR "online intervention*" OR eHealth OR mHealth OR "mobile app intervention*" OR "smartphone app intervention*" OR "mobile phone intervention*" OR "text messag* intervention*" OR "SMS intervention*" OR "wearable electronic device*" OR "activity tracker*" OR "fitness tracker*" OR Fitbit* OR pedometer*) ) |  |  |
|  | #3 | #1 AND #2 |  |  |
| PubMed | #1 | ( "physical activity"[ti] OR walking[ti] OR steps[ti] OR "step count"[ti] OR "step counts"[ti] OR sedentary[ti] OR "sedentary behavior"[ti] OR "sedentary behaviour"[ti] OR sitting[ti] ) | 4485 | 2026/05/03 |
|  | #2 | ( "digital behavior"[tiab] OR "digital behaviour"[tiab] OR "digital intervention"[tiab] OR "digital interventions"[tiab] OR "web-based intervention"[tiab] OR "web-based interventions"[tiab] OR "internet-based intervention"[tiab] OR "internet-based interventions"[tiab] OR "online intervention"[tiab] OR "online interventions"[tiab] OR eHealth[tiab] OR mHealth[tiab] OR "mobile app intervention"[tiab] OR "mobile app interventions"[tiab] OR "smartphone app intervention"[tiab] OR "smartphone app interventions"[tiab] OR "mobile phone intervention"[tiab] OR "mobile phone interventions"[tiab] OR "text message intervention"[tiab] OR "text message interventions"[tiab] OR "SMS intervention"[tiab] OR "SMS interventions"[tiab] OR "wearable electronic device"[tiab] OR "wearable electronic devices"[tiab] OR "activity tracker"[tiab] OR "activity trackers"[tiab] OR "fitness tracker"[tiab] OR "fitness trackers"[tiab] OR Fitbit[tiab] OR pedometer[tiab] OR pedometers[tiab] ) |  |  |
|  | #3 | #1 AND #2 |  |  |

Table 2. Characteristics of the included studies and their contribution to the primary quantitative synthesis, qualitative synthesis, or sensitivity analyses.

| Author | Sample size (sex) | Age (years) | Participant characteristics | Experimental duration | Control digital component | Control PA content | Experimental digital component | Experimental PA content |
| --- | --- | --- | --- | --- | --- | --- | --- | --- |
| Unick, Duffy (1) | 46; 42 F, 4 M | 55.2 ± 8.3 | Clinical population: cancer survivors | Intervention: 12 weeks  follow-up: 6 months | No structured digital behavior change intervention | PA education newsletters | Web-based intervention | Automated weekly lessons, homework, exercise planning/logging, progressive MVPA goals, and tailored feedback |
| Cheung, Kwok (2) | 98; 85 F, 13 M | 54.5 ± 7.6 | Clinical population: cancer survivors | Intervention: 10 weeks  follow-up: 3 months postintervention | No structured digital behavior change intervention | Written PA recommendations | App-based intervention | Automated weekly M-PAC–based lessons and PA promotion content |
| Collombon, Bolman (3) | 954; mostly female | 59.4 ± 6.4 | Adults | Intervention: 12 weeks  follow-up: 6 months | No structured digital behavior change intervention | No active PA support | Web-based intervention with mobile components | Computer-based PA intervention supplemented with activity tracker, ecological momentary intervention, or chatbot |
| Fichtner, Tinsel (4) | 2330; ~75% F | ≈43.0 | Adults | Intervention: 12 weeks  follow-up: 6 and 12 months | No structured digital behavior change intervention | Static evidence-based PA information | Web-based intervention | Interactive personalized online PA coaching, activity planning, tailored feedback, and prompts |
| Joseph, Todd (5) | 60; 60 F | 38.4 ± 6.9 | Adults with obesity | Intervention: 4 months  follow-up: 8 months | No structured digital PA behavior change intervention | General wellness content | App-based intervention | Culturally tailored PA modules, Fitbit-integrated self-monitoring, text messages, discussion boards, and PA goal setting |
| Lugones-Sanchez, Recio-Rodriguez (6) | 650; 445 F, 205 M | ≈48.3 | Adults with overweight/obesity | Intervention: 3 months  follow-up: 12 months | No structured digital behavior change intervention | Brief counseling on healthy diet and PA | App- and wearable-based intervention | Smartphone app plus smart band for lifestyle self-monitoring and PA promotion |
| Myers, McMahon (7) | 461; 301 F, 160 M | 41.9 ± 10.9 | Adults with overweight/obesity | Intervention: 30 days  follow-up: 60 days | No structured digital behavior change intervention | Usual care during the study period | Web-based intervention | Self-efficacy–based behavioral challenges designed to promote PA regulation |
| Van Dyck, Herman (8) | 72; 35 F, 37 M | 70.9 ± 4.1 | Adults | Intervention: 5 weeks  follow-up: 3 months | No structured digital behavior change intervention | No active PA support | Web-based intervention | Self-regulation-based PA intervention targeting preintentional and postintentional determinants |
| Miragall, Domínguez-Rodríguez (9) | 76; 85.5% F | 22.2 ± 3.7 | Adults | Intervention: 3 weeks  follow-up: 3 months | No structured digital behavior change intervention | No active PA support | Web- and pedometer-based intervention | Internet-based motivational intervention with individualized PA goals and pedometer-supported step feedback |
| Van Hoye, Wijtzes (10) | 227; 124 F, 103 M | 40.7–44.3 across groups | Adults | Intervention: 4 weeks  follow-up: 3, 6, and 12 months | No structured digital behavior change intervention | PA measurement only or minimal feedback | Wearable feedback intervention | Pedometer or display-based feedback on steps, MVPA, and energy expenditure; coaching arm not included in the primary quantitative synthesis. |
| Kanera, Willems (11) | 462; 369 F, 93 M | 55.9 | Clinical population: cancer survivors | Intervention: 6 months  follow-up: 12 months | No structured digital behavior change intervention | Delayed access after follow-up | Web-based intervention | Fully automated computer-tailored cancer aftercare modules targeting moderate PA and lifestyle behavior |
| Duan, Wienert (12) | 493; mixed sex | ≈19.2 | Adults | Intervention: 8 weeks  follow-up: 1 month | No structured digital behavior change intervention | No active PA support | Web-based intervention | HAPA-based PA and fruit/vegetable intake modules; first 4 weeks focused on PA |
| Strachan, Marcotte (13) | 244; 82.4% F | 29.6 ± 10.8 | Adults | Intervention: brief online task  follow-up: 4 and 8 weeks | No structured digital behavior change intervention | No structured PA behavior-change strategy | Web-based intervention | Online physically active possible-self task, with or without self-regulatory strategy formation |
| Maher, Ferguson (14) | 110; 78 F, 32 M | 35.6 ± 12.4 | Adults | Intervention: 50 days  follow-up: 8 and 20 weeks | No structured digital behavior change intervention | Wait-list control with no active PA support | Social-networking app-based intervention | Facebook app with pedometers, self-monitoring, team-based social support, gamification, and walking/MVPA promotion |
| Irvine, Gelatt (15) | 368; 69% F | 60.3 ± 4.9 | Adults | Intervention: 12 weeks  follow-up: 6 months | No structured digital behavior change intervention | No active PA support | Web-based intervention | Tailored exercise planning across endurance, flexibility, strengthening, and balance, with weekly video/text support and barrier problem-solving |
| Bossen, Veenhof (16) | 199; 129 F, 70 M | 62.0 ± 5.7 | Clinical population: knee and/or hip osteoarthritis | Intervention: 9 weeks  follow-up: 3 and 12 months | No structured digital behavior change intervention | Wait-list control with no active PA support | Web-based intervention | Fully automated graded PA program based on the participant’s preferred recreational activity |
| Peels, Bolman (17) | 2140; mixed sex | >50 years | Adults | Intervention: 4 months; follow-up: 12 months | No structured digital behavior change intervention | Wait-list control with no tailored PA advice | Web-based tailored intervention | Tailored PA advice targeting PA awareness, psychosocial factors, self-regulation, and local PA opportunities. |

Notes. F = female; M = male; PA = physical activity; MVPA = moderate-to-vigorous physical activity; M-PAC = multi-process action control; HAPA = Health Action Process Approach. Seventeen studies were included in the review. Thirteen studies contributed to the primary quantitative synthesis. Unick et al. and Duan et al. were retained in the qualitative synthesis because compatible numerical data for the prespecified primary effect-size extraction were not available. Joseph et al. was examined in sensitivity analysis because it used an active digital wellness comparator. Myers et al. was examined in sensitivity analysis because the available estimate was an adjusted path-model direct effect rather than a conventional between-group PA effect. Peels et al. contributed the web-based basic arm to the primary model, and the alternative web-based environmental arm was examined in sensitivity analysis.

Table 3. Meta-regression analyses of attenuation and intervention duration.

| **Analysis** | **Moderator** | **beta** | **95% CI** | **p value** |
| --- | --- | --- | --- | --- |
| Attenuation analysis including all time points | Follow-up duration after intervention end, months | -0.001 | -0.02 to 0.01 | 0.790 |
| Attenuation analysis excluding post-intervention assessments | Follow-up duration after intervention end, months | -0.01 | -0.04 to 0.01 | 0.196 |
| Intervention duration analysis | Intervention duration, weeks | -0.003 | -0.02 to 0.02 | 0.740 |
| Adjusted attenuation model | Follow-up duration after intervention end, months | -0.0004 | -0.01 to 0.01 | 0.846 |
| Adjusted attenuation model | Intervention duration, weeks | -0.008 | -0.04 to 0.03 | 0.510 |

Note. Post-intervention assessments were coded as 0 months after intervention end. Beta values represent the change in Hedges’ g per unit increase in the moderator. Time-course analyses used multilevel models with cluster-robust variance estimation when multiple effect sizes were contributed by the same study.

Table 4. Exploratory subgroup analyses based on the 13 studies contributing to the primary quantitative synthesis.

| **Subgroup analysis** | **Category** | **Effects/studies** | **Hedges' g** | **95% CI** | **p value** | **I2 (%)** | **p for subgroup difference** |
| --- | --- | --- | --- | --- | --- | --- | --- |
| Population type | General/non-clinical adults | 9/9 | 0.15 | -0.05 to 0.35 | 0.116 | 72.5 | 0.622 |
| Population type | Clinical or health-risk populations | 4/4 | 0.08 | -0.19 to 0.35 | 0.431 | 48.3 | 0.622 |
| Measurement method | Self-reported physical activity | 7/7 | 0.15 | -0.02 to 0.32 | 0.075 | 72.4 | 0.618 |
| Measurement method | Device-measured physical activity | 6/6 | 0.09 | -0.25 to 0.44 | 0.516 | 52.6 | 0.618 |

Note. Effects/studies indicates the number of effect sizes and the number of unique studies contributing to each analysis. Subgroup analyses were exploratory and should be interpreted cautiously because of the limited number of studies within categories.

Table 5. Sensitivity analyses.

| **Analysis** | **Effects/studies** | **Hedges' g** | **95% CI** | **p value** | **I2 (%)** | **95% prediction interval** |
| --- | --- | --- | --- | --- | --- | --- |
| Primary model | 13/13 | 0.13 | -0.01 to 0.26 | 0.066 | 64.2 | -0.23 to 0.48 |
| Alternative Peels web-based environmental arm | 13/13 | 0.12 | -0.01 to 0.26 | 0.070 | 64.2 | -0.23 to 0.48 |
| Including active digital comparator study | 14/14 | 0.12 | -0.02 to 0.25 | 0.085 | 62.7 | -0.23 to 0.47 |
| Including adjusted path-model direct effect | 14/14 | 0.12 | -0.00 to 0.24 | 0.054 | 60.2 | -0.20 to 0.43 |
| Excluding brief one-session intervention | 12/12 | 0.11 | -0.03 to 0.25 | 0.123 | 62.8 | -0.24 to 0.45 |

Note. The primary model included 13 studies with compatible effect sizes. The active digital comparator sensitivity analysis added Joseph et al.; the adjusted path-model direct-effect sensitivity analysis added Myers et al.; and the alternative Peels analysis replaced the web-based basic arm with the web-based environmental arm. Peels et al. was therefore not excluded from the primary model.

Table 6. Small-study-effect analysis based on the 13 studies contributing to the primary quantitative synthesis.

| **Test** | **Test statistic** | **p value** |
| --- | --- | --- |
| Egger's regression test | 0.91 | 0.383 |

Note. Egger's regression test was based on the 13 studies contributing to the primary quantitative synthesis and should be interpreted cautiously because of the limited number of studies; a non-significant result should not be interpreted as definitive evidence that publication bias or small-study effects are absent.

Table 7. Post-intervention access, engagement reporting, intervention modality, and intervention duration of included studies.

| **Author** | **Digital modality and active intervention period** | **Follow-up after active intervention end** | **Continued digital access or structured support after intervention end** | **Engagement/adherence metrics reported** | **Source trace and durability interpretation** |
| --- | --- | --- | --- | --- | --- |
| Unick, Duffy (1) | Web-based Energize! program; 12 weeks | 6 months after baseline; 3-month no-contact follow-up after the postintervention assessment | No active contact during follow-up was reported; continued access to the study website after week 12 was not reported/unclear. | Video lessons viewed, exercise plans submitted, homework assignments completed, exercise-minutes logging, weekly goal achievement, retention, and satisfaction were reported. | Original article describes a 12-week website program, a no-contact follow-up, and detailed engagement percentages. The estimate reflects durability after active weekly content and feedback ended, but complete digital withdrawal cannot be confirmed. |
| Cheung, Kwok (2) | Smartphone WExercise app; 10 weekly PA lessons plus introductory/safety lessons | 12 weeks postintervention | Continued app access, reminders, or new automated content after completion of the weekly lessons was not reported/unclear. | App usage was summarized; completion of >=75% of lessons and lesson completion from first to last lesson were reported. Instant-message reminders were used during lesson delivery. | Original article describes individualized app access, weekly automated lessons, app usage statistics, and reminders during delivery. The follow-up is after scheduled app delivery, but postintervention access was not clearly specified. |
| Collombon, Bolman (3) | Web-based intervention with added mobile elements (activity tracker, ecological momentary intervention, or chatbot); 12 weeks | Approximately 23/24 weeks after baseline; about 11/12 weeks after the 12-week intervention period | Continued access to the computer-based intervention, activity tracker/app, ecological momentary messages, or chatbot after the 12-week period was not reported/unclear. | Dropout was reported. Usability and appreciation were assessed but described as reported elsewhere; detailed maintenance-period engagement metrics were not consistently available in the article. | Original article describes AP/IM sessions and added mobile elements during 12 weeks, followed by later assessment. Durability should be interpreted as follow-up after scheduled delivery, not confirmed complete withdrawal of all digital components. |
| Fichtner, Tinsel (4) | Interactive web-based TK-FitnessCoach; recommended use for 12 weeks | 6 and 12 months after the program conclusion | Continued access to the interactive web program or prompts after the 12-week recommended-use period was not reported/unclear. | Weekly login behavior was used to classify users (constant, halftime, partial, rare, one-time), and associations with PA were examined. | Original article specifies T1 as 12-week postintervention and T2/T3 as 6- and 12-month follow-ups after program conclusion. Login-based engagement is available, but complete withdrawal of web access was not established. |
| Joseph, Todd (5) | Smartphone Smart Walk app with Fitbit-integrated self-monitoring, discussion boards, and text messages; 4 months | 8 months after baseline; months 5-8 were a no-contact follow-up | Participants retained access to their assigned smartphone app during the no-contact follow-up, but no new content was delivered. PA-promotion text messages were delivered during the active intervention period. | Retention, Smart Walk app use, Fitbit wear, text-message delivery, and satisfaction were reported. | Original article explicitly states that months 5-8 were no-contact and that app access continued without new content. This study was examined in sensitivity analysis because it used an active digital wellness comparator. |
| Lugones-Sanchez, Recio-Rodriguez (6) | Smartphone app plus smart band; 3 months | 12 months after randomization; approximately 9 months after return of the digital tools | Participants returned the intervention smartphone and smart band at 3 months; thereafter they did not have access to the intervention devices and were advised not to use other digital weight-loss tools until study end. | Adherence to app self-monitoring was assessed by days with app records; sufficient adherence was defined by app use for >60 days during the 3-month access period. Retention was also reported. | Original article explicitly reports return of tools and loss of access after 3 months. This is the clearest example of follow-up after withdrawal of study-provided digital tools. |
| Myers, McMahon (7) | Web-based Fun for Wellness intervention; 30 days of 24-hour access | 60 days after baseline; about 30 days after the 30-day access period | The intervention group had 30 days of 24-hour access; continued intervention access after day 30 was not reported/unclear. Usual-care participants received delayed access after data collection closed. | Challenge completion was tracked by software and used for an engagement scoring system; participation points and engagement threshold were defined. | Original article specifies access from T1 to T2 and a T3 survey after the access period. This study was examined in sensitivity analysis because the available estimate was an adjusted path-model direct effect. |
| Van Dyck, Herman (8) | Web-based MyPlan 2.0; 5 consecutive weekly website visits | 3 months after baseline; after completion of the 5-week intervention | Continued access to web modules, email reminders, or telephone reminders after the 5 scheduled website visits was not reported/unclear. | Weekly website visits and reminders were described, but detailed maintenance-period engagement/adherence metrics were not clearly reported. | Original article describes 5 website visits, weekly email reminders, and phone reminders during delivery. Follow-up occurred after scheduled delivery, but complete digital withdrawal cannot be confirmed. |
| Miragall, Domínguez-Rodríguez (9) | Internet-based motivational intervention, with or without Fitbit/pedometer support; 3 weeks | 3 months after the 3-week intervention; participants wore the pedometer again for 1 week at follow-up measurement | Participants returned the pedometer after 3 weeks. Continued access to the one-session internet program or Fitbit website after the intervention was not reported/unclear. | Objective step outcomes were collected; detailed website engagement metrics were limited/not reported. | Original article states that intervention participants accessed the web intervention once, wore pedometers for 3 weeks, returned the pedometer, and wore it again only for follow-up measurement. Ongoing digital support after intervention was not clearly described. |
| Van Hoye, Wijtzes (10) | Wearable feedback intervention using pedometer/display feedback; 4 weeks | 1 week, 3 months, 6 months, and 12 months after the 4-week intervention | Participants turned in the feedback device after the intervention. Follow-up assessments used a sensor without feedback; repeated assessments and reports may nevertheless have served as reinforcement. | Wear-time/compliance and retention were reported; adherence to the intended treatment was considered in analyses. | Original article explicitly reports return of feedback devices and no-feedback sensors at follow-ups. Thus, this largely reflects withdrawal of wearable feedback, although assessment procedures/reports may have acted as boosters. |
| Kanera, Willems (11) | Web-based computer-tailored cancer aftercare program; 6 months | 12 months after baseline; after the main 6-month intervention period | Control participants received delayed access after follow-up. Continued access for intervention participants after 6 months was not reported/unclear. | Program logging data were used to determine whether the PA module was used; automatic email reminders invited questionnaire/module completion. | Original article describes online modules, automatic reminders, program logging, and module-use moderator analysis. Follow-up occurred after scheduled delivery, but continued access among intervention participants was uncertain. |
| Duan, Wienert (12) | Web-based HAPA intervention; 8 weeks overall, with PA emphasized during weeks 1-4 | 1 month after the 8-week intervention | Continued access to web modules, reminders, or feedback after intervention completion was not reported/unclear. | Email links, text-message reminders, and verbal reminders were used during the weekly intervention and data collection; dropout and low engagement were discussed, but detailed module-use metrics were limited/not reported. | Original article describes weekly web sessions and reminders through the intervention/measurement period. This study was retained in qualitative synthesis because compatible numerical data for the primary effect-size extraction were unavailable. |
| Strachan, Marcotte (13) | Brief online physically active possible-self task; single session | 4 and 8 weeks after completing the online task | No ongoing digital tool, automated feedback, reminders, or structured digital support after the online task was reported. | Task completion and follow-up survey completion were reported; no ongoing engagement metrics were applicable. | Original article describes a one-time online intervention task followed by emailed follow-up surveys. This represents post-task follow-up rather than maintenance after a sustained digital program. |
| Maher, Ferguson (14) | Facebook Active Team app with pedometer-supported self-monitoring, team social support, and gamification; 50 days | 8 weeks and 20 weeks after baseline; 20 weeks was about 3 months after intervention completion | Continued app or pedometer access after the 50-day challenge was not reported/unclear. Control teams received delayed access to the app and pedometer after study completion. | App usage statistics were reported, including visits, step-logging, virtual gifts, wall posts, and log-in dosage. | Original article describes 50-day app/pedometer access, wait-list delayed access for controls, and detailed usage statistics. Continued access after the challenge was not clearly specified. |
| Irvine, Gelatt (15) | Web-based Active After 55; 12 weekly sessions | 6 months after baseline; 12 weeks after the postintervention assessment | Participants still completing sessions after the first follow-up were encouraged to continue until all sessions were completed; continued access after completing all sessions was not reported/unclear. Control participants received delayed access after T3. | Program use, session completion, website visits, time on site, segment access, attrition, retention, and dose-response information were reported. | Original article explicitly allows continued completion after T2 for late users. Thus, some follow-up may include extended intervention exposure rather than complete withdrawal for all participants. |
| Bossen, Veenhof (16) | Web-based graded PA program (Join2move); 9 weeks | 3 and 12 months after baseline | Continued intervention access after the 9-week program was not reported/unclear. Waiting-list controls received access after the follow-up period. | Program usage was measured by weekly modules completed; starting the program, module completion, and the adherence threshold of 6 of 9 modules were reported. | Original article describes weekly modules, automated emails during delivery, no human support, and program-usage metrics. Postintervention access for intervention participants was not clearly specified. |
| Peels, Bolman (17) | Web-based tailored Active Plus intervention; three tailored advice moments over 4 months | 12 months after baseline; approximately 8 months after intervention completion | Continued access to the Active Plus website after the 4-month tailored advice period was not reported/unclear. Participants also received emailed PDF copies of their tailored advice. | Website use/appreciation and saving of materials were discussed, but detailed postintervention engagement metrics were limited/not clearly reported. | Original article describes web log-in, three advice moments, emailed PDF advice, and discussion of website use/appreciation. The primary model used the web-based basic arm; complete withdrawal of web/advice materials cannot be confirmed. |

Note. PA = physical activity; DBCI = digital behavior change intervention; NR = not reported. Active intervention end was operationalized as the scheduled end of active structured DBCI delivery in each trial. Information in this table was extracted from the original included articles and cross-checked against Supplementary Table 2. Continued digital access or structured support refers to access to study-provided websites, apps, wearable feedback, automated reminders, digital feedback, or structured digital support after active delivery ended. When the original article did not clearly state postintervention access or engagement metrics, information was coded as not reported/unclear. This table clarifies whether follow-up effects represent durability after the end of active structured delivery, complete withdrawal of digital tools, or an unclear intermediate situation.

References

1.	Unick JL, Duffy C, Dizon D, Fenton MA, Cao Z, Oselinsky K, et al. Evaluation of a Translatable Web-Based Intervention for Increasing Physical Activity among Cancer Survivors: Pilot Randomized Trial. *JMIR cancer* (2025) 11:e79610.

2.	Cheung DST, Kwok TWH, Liu S, Rhodes RE, Chau PH, Chiang C-L, et al. A Smartphone App (Wexercise) to Promote Physical Activity among Cancer Survivors: Randomized Controlled Trial. *Journal of Medical Internet Research* (2025) 27:e75839.

3.	Collombon EH, Bolman CA, de Bruijn GJ, Peels DA, Verboon P, Lechner L. The Efficacy of Online Physical Activity Interventions with Added Mobile Elements within Adults Aged 50 Years and Over: Randomized Controlled Trial. *Applied Psychology: Health and Well‐Being* (2024) 16(4):1921-43.

4.	Fichtner UA, Tinsel I, Sehlbrede M, Maiwald P, Bischoff M, Metzner G, et al. Effects of a Digital Intervention on Physical Activity in Adults: A Randomized Controlled Trial in a Large-Scale Sample. *Internet Interventions* (2024) 37:100762.

5.	Joseph RP, Todd M, Ainsworth BE, Vega-López S, Adams MA, Hollingshead K, et al. Smart Walk: A Culturally Tailored Smartphone-Delivered Physical Activity Intervention for Cardiometabolic Risk Reduction among African American Women. *International journal of environmental research and public health* (2023) 20(2):1000.

6.	Lugones-Sanchez C, Recio-Rodriguez JI, Agudo-Conde C, Repiso-Gento I, G Adalia E, Ramirez-Manent JI, et al. Long-Term Effectiveness of a Smartphone App Combined with a Smart Band on Weight Loss, Physical Activity, and Caloric Intake in a Population with Overweight and Obesity (Evident 3 Study): Randomized Controlled Trial. *Journal of medical Internet research* (2022) 24(2):e30416.

7.	Myers ND, McMahon A, Prilleltensky I, Lee S, Dietz S, Prilleltensky O, et al. Effectiveness of the Fun for Wellness Web-Based Behavioral Intervention to Promote Physical Activity in Adults with Obesity (or Overweight): Randomized Controlled Trial. *JMIR formative research* (2020) 4(2):e15919.

8.	Van Dyck D, Herman K, Poppe L, Crombez G, De Bourdeaudhuij I, Gheysen F. Results of Myplan 2.0 on Physical Activity in Older Belgian Adults: Randomized Controlled Trial. *Journal of medical Internet research* (2019) 21(10):e13219.

9.	Miragall M, Domínguez-Rodríguez A, Navarro J, Cebolla A, Baños RM. Increasing Physical Activity through an Internet-Based Motivational Intervention Supported by Pedometers in a Sample of Sedentary Students: A Randomised Controlled Trial. *Psychology & health* (2018) 33(4):465-82.

10.	Van Hoye K, Wijtzes AI, Lefevre J, De Baere S, Boen F. Year-Round Effects of a Four-Week Randomized Controlled Trial Using Different Types of Feedback on Employees’ Physical Activity. *BMC Public Health* (2018) 18(1):492.

11.	Kanera IM, Willems RA, Bolman CA, Mesters I, Verboon P, Lechner L. Long-Term Effects of a Web-Based Cancer Aftercare Intervention on Moderate Physical Activity and Vegetable Consumption among Early Cancer Survivors: A Randomized Controlled Trial. *International Journal of Behavioral Nutrition and Physical Activity* (2017) 14(1):19.

12.	Duan YP, Wienert J, Hu C, Si GY, Lippke S. Web-Based Intervention for Physical Activity and Fruit and Vegetable Intake among Chinese University Students: A Randomized Controlled Trial. *Journal of medical Internet research* (2017) 19(4):e106.

13.	Strachan SM, Marcotte MM, Giller TM, Brunet J, Schellenberg BJ. An Online Intervention to Increase Physical Activity: Self-Regulatory Possible Selves and the Moderating Role of Task Self-Efficacy. *Psychology of Sport and Exercise* (2017) 31:158-65.

14.	Maher C, Ferguson M, Vandelanotte C, Plotnikoff R, De Bourdeaudhuij I, Thomas S, et al. A Web-Based, Social Networking Physical Activity Intervention for Insufficiently Active Adults Delivered Via Facebook App: Randomized Controlled Trial. *Journal of medical Internet research* (2015) 17(7):e174.

15.	Irvine AB, Gelatt VA, Seeley JR, Macfarlane P, Gau JM. Web-Based Intervention to Promote Physical Activity by Sedentary Older Adults: Randomized Controlled Trial. *Journal of medical Internet research* (2013) 15(2):e19.

16.	Bossen D, Veenhof C, Van Beek KE, Spreeuwenberg PM, Dekker J, De Bakker DH. Effectiveness of a Web-Based Physical Activity Intervention in Patients with Knee and/or Hip Osteoarthritis: Randomized Controlled Trial. *Journal of medical Internet research* (2013) 15(11):e2662.

17.	Peels DA, Bolman C, Golsteijn RHJ, de Vries H, Mudde AN, van Stralen MM, et al. Long-Term Efficacy of a Printed or a Web-Based Tailored Physical Activity Intervention among Older Adults. *International Journal of Behavioral Nutrition and Physical Activity* (2013) 10(1):104.
